# 2-Oxo-2-(2-thien­yl)acetic acid

**DOI:** 10.1107/S160053681004403X

**Published:** 2010-11-06

**Authors:** Guy Crundwell

**Affiliations:** a Department of Chemistry and Biochemistry, Central Connecticut State University, 1619 Stanley Street, New Britain, CT 06053, USA

## Abstract

The structure of the title compound, C_6_H_4_O_3_S, displays inter­molecular hydrogen-bonding dimers. The structure exhibits a thienyl-ring flip disorder of the main mol­ecule [occupancy ratio = 91.3 (2):8.7 (2)].

## Related literature

For a discussion of ring-flip disorder in unsubstituted 2- and 3-thienyl rings, see: Crundwell *et al.* (2003 ▶). For information on simple O—H⋯O interactions, see: Bernstein *et al.* (1995 ▶).
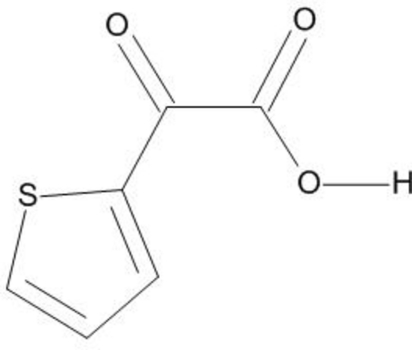


## Experimental

### 

#### Crystal data

C_6_H_4_O_3_S
*M* *_r_* = 156.15
Monoclinic, 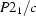

*a* = 3.7481 (10) Å
*b* = 15.314 (3) Å
*c* = 10.727 (3) Å
β = 93.30 (2)°
*V* = 614.7 (3) Å^3^
*Z* = 4
Mo *K*α radiation
μ = 0.46 mm^−1^
*T* = 293 K
0.34 × 0.21 × 0.11 mm

#### Data collection

Oxford Diffraction Xcalibur Sapphire3 diffractometer
Absorption correction: multi-scan (*CrysAlis PRO*; Oxford Diffraction, 2009 ▶) *T*_min_ = 0.944, *T*_max_ = 1.000
6475 measured reflections
1927 independent reflections
1512 reflections with *I* > 2σ(*I*)
*R*_int_ = 0.032

#### Refinement

*R*[*F* ^2^ > 2σ(*F* ^2^)] = 0.037
*wR*(*F* ^2^) = 0.106
*S* = 1.09
1927 reflections
104 parameters
12 restraints
H-atom parameters constrained
Δρ_max_ = 0.51 e Å^−3^
Δρ_min_ = −0.29 e Å^−3^

### 

Data collection: *CrysAlis PRO* (Oxford Diffraction, 2009 ▶); cell refinement: *CrysAlis PRO*; data reduction: *CrysAlis PRO*; program(s) used to solve structure: *SHELXS97* (Sheldrick, 2008 ▶); program(s) used to refine structure: *SHELXL97* (Sheldrick, 2008 ▶); molecular graphics: *ORTEP-3* (Farrugia, 1997 ▶) and *PLATON* (Spek, 2009 ▶); software used to prepare material for publication: *SHELXTL* (Sheldrick, 2008 ▶).

## Supplementary Material

Crystal structure: contains datablocks I, global. DOI: 10.1107/S160053681004403X/bx2317sup1.cif

Structure factors: contains datablocks I. DOI: 10.1107/S160053681004403X/bx2317Isup2.hkl

Additional supplementary materials:  crystallographic information; 3D view; checkCIF report

## Figures and Tables

**Table 1:** Hydrogen-bond geometry (Å, °)

*D*—H⋯*A*	*D*—H	H⋯*A*	*D*⋯*A*	*D*—H⋯*A*
O1—H1⋯O2^i^	0.82	1.82	2.637 (2)	176

Symmetry code: (i) 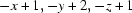.

**Fig. 1.**
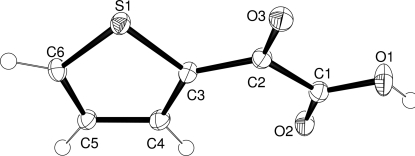
A view of the title compound (Farrugia, 1997). Displacement ellipsoids are drawn at the 50% probability level.

## supplementary crystallographic information

### Comment

The structure of 2-oxo-2-(2-thienyl)acetic acid, C_6_H_4_O_3_S, has monoclinic (*P*2_1_/*c*) symmetry. The structure displays intermolecular hydrogen bonding dimers. The structure exhibits a thienyl-ring flip disorder of the main molecule.

The structure of the title compound displays centrosymmetric *R*_2_^2^(8) dimers by a simple O—H···O interactions (Bernstein *et al.*, 1995). The structure exhibits a thienyl-ring flip disorder of the main molecule with occupancy ratios of 91.3 (2)% to 8.7 (2)%.

### Experimental

The title compound was purchased as 2-thiopheneglyoxylic acid from Aldrich (95% purity). Crystals for this *x*-ray diffraction study were harvested from methanol during routine recrystallization.

### Refinement

During refinement, the thienyl ring showed evidence of ring-flip disorder which is common for unsubstituted 2- and 3-thienyl rings (Crundwell *et al.*, 2003). After finding three of the flipped disordered atoms in the difference map, the rest of the ring was generated and modeled. The final model suggested that the thienyl ring disorder was 8.7 (2)%.

Hydrogen atoms on carbons were included in calculated positions with a C—H distance of 0.93 Å and were included in the refinement in riding motion approximation with *U*_iso_ = 1.2*U*_eq_ of the carrier atom.

The hydroxyl hydrogen was included in a calculated position with a O—H distance of 0.82 Å and was included in the refinement in riding motion approximation with *U*_iso_ = 1.2*U*_eq_ of the carrier atom.

### Crystal data

**Table:**

C_6_H_4_O_3_S	*F*(000) = 320
*M**_r_* = 156.15	*D*_x_ = 1.687 Mg m^−^^3^
Monoclinic, *P*2_1_/*c*	Melting point: 361 K
Hall symbol: -P 2ybc	Mo *K*α radiation, λ = 0.71073 Å
*a* = 3.7481 (10) Å	Cell parameters from 6632 reflections
*b* = 15.314 (3) Å	θ = 3.8–32.0°
*c* = 10.727 (3) Å	µ = 0.46 mm^−^^1^
β = 93.30 (2)°	*T* = 293 K
*V* = 614.7 (3) Å^3^	Plate, yellow
*Z* = 4	0.34 × 0.21 × 0.11 mm

### Data collection

**Table:**

Oxford Diffraction Xcalibur Sapphire3 diffractometer	1927 independent reflections
Radiation source: Enhance (Mo) X-ray Source	1512 reflections with *I* > 2σ(*I*)
graphite	*R*_int_ = 0.032
Detector resolution: 16.1790 pixels mm^-1^	θ_max_ = 32.0°, θ_min_ = 3.8°
ω scans	*h* = −5→5
Absorption correction: multi-scan (*CrysAlis PRO*; Oxford Diffraction, 2009)	*k* = −22→16
*T*_min_ = 0.944, *T*_max_ = 1.000	*l* = −15→15
6475 measured reflections	

### Refinement

**Table:**

Refinement on *F*^2^	Primary atom site location: structure-invariant direct methods
Least-squares matrix: full	Secondary atom site location: difference Fourier map
*R*[*F*^2^ > 2σ(*F*^2^)] = 0.037	Hydrogen site location: inferred from neighbouring sites
*wR*(*F*^2^) = 0.106	H-atom parameters constrained
*S* = 1.09	*w* = 1/[σ^2^(*F*_o_^2^) + (0.0678*P*)^2^ + 0.018*P*] where *P* = (*F*_o_^2^ + 2*F*_c_^2^)/3
1927 reflections	(Δ/σ)_max_ = 0.002
104 parameters	Δρ_max_ = 0.51 e Å^−^^3^
12 restraints	Δρ_min_ = −0.29 e Å^−^^3^

### Special details

**Table:**

Experimental. Hydrogen atoms on carbons were included in calculated positions with a C—H distance of 0.93 Å and were included in the refinement in riding motion approximation with *U*_iso_ = 1.2*U*_eq_ of the carrier atom.The hydroxyl hydrogen was included in a calculated position with a O—H distance of 0.82 Å and was included in the refinement in riding motion approximation with *U*_iso_ = 1.2*U*_eq_ of the carrier atom.
Geometry. All e.s.d.'s (except the e.s.d. in the dihedral angle between two l.s. planes) are estimated using the full covariance matrix. The cell e.s.d.'s are taken into account individually in the estimation of e.s.d.'s in distances, angles and torsion angles; correlations between e.s.d.'s in cell parameters are only used when they are defined by crystal symmetry. An approximate (isotropic) treatment of cell e.s.d.'s is used for estimating e.s.d.'s involving l.s. planes.
Refinement. Refinement of *F*^2^ against ALL reflections. The weighted *R*-factor *wR* and goodness of fit *S* are based on *F*^2^, conventional *R*-factors *R* are based on *F*, with *F* set to zero for negative *F*^2^. The threshold expression of *F*^2^ > σ(*F*^2^) is used only for calculating *R*-factors(gt) *etc*. and is not relevant to the choice of reflections for refinement. *R*-factors based on *F*^2^ are statistically about twice as large as those based on *F*, and *R*- factors based on ALL data will be even larger.

### Fractional atomic coordinates and isotropic or equivalent isotropic displacement parameters (Å^2^)

**Table:**

	*x*	*y*	*z*	*U*_iso_*/*U*_eq_	Occ. (<1)
O1	0.1858 (3)	0.98317 (6)	0.36658 (10)	0.0261 (3)	
H1	0.2759	1.0208	0.4128	0.039*	
O2	0.5095 (3)	0.89316 (7)	0.49326 (9)	0.0207 (2)	
C1	0.2916 (4)	0.90590 (8)	0.40596 (12)	0.0167 (3)	
C2	0.1123 (4)	0.83170 (8)	0.32857 (12)	0.0156 (3)	
O3	−0.0839 (3)	0.85113 (7)	0.23733 (9)	0.0207 (2)	
C3	0.1813 (3)	0.74199 (8)	0.36784 (12)	0.0153 (3)	0.9131 (17)
C4	0.3362 (8)	0.70703 (19)	0.4767 (2)	0.0176 (4)	0.9131 (17)
H4	0.4368	0.7412	0.5413	0.021*	0.9131 (17)
C5	0.3282 (10)	0.61491 (14)	0.4812 (2)	0.0158 (3)	0.9131 (17)
H5	0.4176	0.5815	0.5484	0.019*	0.9131 (17)
C6	0.1687 (5)	0.58091 (10)	0.37166 (15)	0.0158 (3)	0.9131 (17)
H6	0.1417	0.5214	0.3564	0.019*	0.9131 (17)
S1	0.02766 (10)	0.65987 (2)	0.26826 (3)	0.01633 (14)	0.9131 (17)
C3B	0.1813 (3)	0.74199 (8)	0.36784 (12)	0.0153 (3)	0.0869 (17)
C4B	0.057 (4)	0.6842 (10)	0.2959 (14)	0.01633 (14)	0.0869 (17)
H4B	−0.0609	0.6947	0.2187	0.020*	0.0869 (17)
C5B	0.122 (6)	0.5982 (11)	0.350 (2)	0.0158 (3)	0.0869 (17)
H5B	0.0539	0.5454	0.3127	0.019*	0.0869 (17)
C6B	0.303 (13)	0.6081 (13)	0.464 (3)	0.0158 (3)	0.0869 (17)
H6B	0.3739	0.5612	0.5151	0.019*	0.0869 (17)
S1B	0.384 (3)	0.7158 (6)	0.5057 (7)	0.0176 (4)	0.0869 (17)

### Atomic displacement parameters (Å^2^)

**Table:**

	*U*^11^	*U*^22^	*U*^33^	*U*^12^	*U*^13^	*U*^23^
O1	0.0349 (6)	0.0124 (4)	0.0291 (6)	0.0006 (4)	−0.0140 (4)	−0.0003 (4)
O2	0.0264 (5)	0.0153 (5)	0.0193 (5)	0.0004 (4)	−0.0080 (4)	−0.0003 (4)
C1	0.0180 (6)	0.0151 (6)	0.0170 (6)	−0.0003 (5)	0.0004 (5)	−0.0007 (5)
C2	0.0166 (6)	0.0150 (6)	0.0152 (6)	0.0009 (5)	−0.0005 (4)	−0.0003 (5)
O3	0.0245 (5)	0.0203 (5)	0.0166 (5)	0.0016 (4)	−0.0060 (4)	0.0009 (4)
C3	0.0159 (6)	0.0132 (6)	0.0165 (6)	0.0006 (5)	−0.0005 (5)	−0.0015 (5)
C4	0.0204 (11)	0.0172 (9)	0.0147 (13)	−0.0002 (7)	−0.0017 (9)	−0.0020 (9)
C5	0.0181 (9)	0.0144 (7)	0.0147 (10)	0.0001 (6)	−0.0009 (7)	−0.0009 (6)
C6	0.0160 (8)	0.0128 (7)	0.0184 (8)	0.0012 (6)	−0.0017 (6)	0.0023 (6)
S1	0.0178 (2)	0.0142 (2)	0.0167 (2)	−0.00080 (14)	−0.00148 (14)	−0.00162 (13)
C3B	0.0159 (6)	0.0132 (6)	0.0165 (6)	0.0006 (5)	−0.0005 (5)	−0.0015 (5)
C4B	0.0178 (2)	0.0142 (2)	0.0167 (2)	−0.00080 (14)	−0.00148 (14)	−0.00162 (13)
C5B	0.0160 (8)	0.0128 (7)	0.0184 (8)	0.0012 (6)	−0.0017 (6)	0.0023 (6)
C6B	0.0181 (9)	0.0144 (7)	0.0147 (10)	0.0001 (6)	−0.0009 (7)	−0.0009 (6)
S1B	0.0204 (11)	0.0172 (9)	0.0147 (13)	−0.0002 (7)	−0.0017 (9)	−0.0020 (9)

### Geometric parameters (Å, °)

**Table:**

O1—C1	1.3102 (16)	C5—C6	1.389 (2)
O1—H1	0.8200	C5—H5	0.9300
O2—C1	1.2223 (16)	C6—S1	1.7041 (15)
C1—C2	1.5387 (19)	C6—H6	0.9300
C2—O3	1.2265 (17)	C4B—C5B	1.452 (16)
C2—C3	1.4558 (18)	C4B—H4B	0.9300
C3—C4	1.382 (3)	C5B—C6B	1.380 (17)
C3—S1	1.7272 (13)	C5B—H5B	0.9300
C4—C5	1.412 (3)	C6B—S1B	1.730 (18)
C4—H4	0.9300	C6B—H6B	0.9300
			
C1—O1—H1	109.5	C6—C5—H5	124.6
O2—C1—O1	124.58 (12)	C4—C5—H5	124.6
O2—C1—C2	123.18 (12)	C5—C6—S1	112.77 (15)
O1—C1—C2	112.23 (11)	C5—C6—H6	123.6
O3—C2—C3	123.25 (12)	S1—C6—H6	123.6
O3—C2—C1	118.33 (12)	C6—S1—C3	91.96 (7)
C3—C2—C1	118.42 (11)	C5B—C4B—H4B	124.7
C4—C3—C2	132.00 (15)	C6B—C5B—C4B	108.5 (16)
C4—C3—S1	110.46 (14)	C6B—C5B—H5B	125.8
C2—C3—S1	117.44 (10)	C4B—C5B—H5B	125.8
C3—C4—C5	114.04 (18)	C5B—C6B—S1B	113.7 (16)
C3—C4—H4	123.0	C5B—C6B—H6B	123.1
C5—C4—H4	123.0	S1B—C6B—H6B	123.1
C6—C5—C4	110.76 (18)		

### Hydrogen-bond geometry (Å, °)

**Table:**

*D*—H···*A*	*D*—H	H···*A*	*D*···*A*	*D*—H···*A*
O1—H1···O2^i^	0.82	1.82	2.637 (2)	176

Symmetry codes: (i) −*x*+1, −*y*+2, −*z*+1.
